# The Prevalence of Transfusion-Transmitted Diseases Among Blood Donors in the Central Blood Bank in Makkah, Saudi Arabia

**DOI:** 10.7759/cureus.48881

**Published:** 2023-11-16

**Authors:** Mohammad Albshri, Palanisamy Manikandan, Mamdouh Allahyani, Abdulelah Aljuaid, Mazen M Almehmadi, Kamal Alzabeedi, Mohamed Babalgaith, Mosa Alghamdi, Faris Alharbi, Mohammed Alhazmi

**Affiliations:** 1 Department of Hematology, The Regional Laboratory, Makkah, SAU; 2 Department of Medical Laboratory Sciences, College of Applied Medical Sciences, Majmaah University, Majmaah, SAU; 3 Department of Clinical Laboratory Sciences, College of Applied Medical Sciences, Taif University, Taif, SAU; 4 Department of Clinical Biochemistry, The Regional Laboratory, Makkah, SAU; 5 Central Blood Bank, General Directorate of Health Affairs, Makkah Region, Makkah, SAU

**Keywords:** serology, nat, hiv, hcv, hbv, blood donors

## Abstract

Background

This study aimed to analyze the health and demographic characteristics of blood donors in Makkah, Saudi Arabia, and assess the prevalence and correlation of two markers related to hepatitis B infection: hepatitis B virus surface antigen (HBsAg) and anti-hepatitis B virus surface antibody (HBsAb).

Materials and methods

The study used a retrospective design and collected data from the Central Blood Bank in Makkah, Saudi Arabia, in 2022. The sample size was 7,875 blood donors. The study used various methods, such as serological testing, nucleic acid testing (NAT), and statistical analysis. The data were analyzed using Pearson correlation to examine the relationships between different variables.

Results

The predominant age group was 29-39 years, accounting for 46.9% of the total donors. In terms of blood types, O+ve was the most common, representing 40.3% of the donors. The investigation into infectious markers revealed overall low levels of reactivity among donors. For HBsAg, a marker of active hepatitis B infection, only 0.36% of the units were reactive. Conversely, the anti-HBsAb, which indicates immunity to hepatitis B, was reactive in 6.83% of the units. The correlation analysis illuminated some critical relationships. The total number of units tested had a statistically significant, albeit weak, positive relationship with HBsAg reactivity, shown by a Pearson correlation coefficient of 0.030 and a p-value of 0.008. Conversely, the total number of units tested and anti-HBsAb reactivity showed a moderate negative correlation, with a Pearson correlation coefficient of -0.437 and a p-value of less than 0.001. However, no significant correlation was identified between HBsAg and anti-HBsAb reactivity, indicating that active infection and immunity status might not be directly linked.

Conclusion

This extensive study provides in-depth insights into the sociodemographic characteristics of blood donors and the prevalence of key infectious markers within this population. It underlines the imperative of rigorous screening of blood units, particularly given the low immunity levels to hepatitis B identified. Also, the study showed the importance of screening blood units and vaccinating people against hepatitis B. It also suggested the need for more research on blood safety and infection-immunity relationships.

## Introduction

Blood transfusion, the process of transferring blood or blood-based products from one person (the donor) into the circulatory system of another person (the recipient), is a fundamental aspect of modern medical practice [1]. It has critical importance across a wide range of medical conditions and procedures. Major surgeries, particularly cardiovascular surgeries and organ transplants, often involve significant blood loss, which necessitates the replacement of lost blood through transfusion [2]. Furthermore, in trauma cases, such as accidents, falls, and gunshot wounds, rapid blood loss can be life-threatening, making blood transfusion a critical component of emergency medical care [3]. Blood transfusions also have crucial roles in the management of chronic illnesses, such as hematologic conditions (severe anemia, thalassemia, or sickle cell disease), which often require regular blood transfusions as part of their long-term management [4]. Similarly, cancer patients undergoing chemotherapy, which can cause severe anemia and thrombocytopenia, frequently require blood transfusions [4]. Despite the undeniable benefits of blood transfusion, it is essential to note that it is not without risks. Transfusion-transmitted infections, including viral infections such as hepatitis B (HBV), hepatitis C (HCV), human immunodeficiency virus (HIV), and human T-lymphotropic virus (HTLV-1), bacterial infections or parasitic infections such as malaria and syphilis, and emerging pathogens such as Zika virus and dengue, are also concerns particularly in regions where the screening of donated blood may not be optimal [5]. These infectious agents constitute a significant risk in the context of blood transfusion, posing potential threats to recipients' health and causing disease or what is known as transfusion-transmitted diseases (TTDs) [5]. The global burden of TTDs is substantial, particularly in resource-limited settings where access to effective donor screening is limited. There are millions of people currently living with HBV, HCV, and HIV worldwide, many of whom may be asymptomatic and could, therefore, unknowingly transmit the disease if they donate blood [6].

Makkah city in Saudi Arabia, owing to its geographic position and unique cultural and religious practices (such as the annual Hajj pilgrimage that attracts millions of visitors), has a distinctive epidemiological profile. The prevalence of TTDs in Saudi Arabia can be influenced by these factors, thereby necessitating a targeted approach to the study and management of these diseases.

The prevalence of TTDs among blood donors in Saudi Arabia is relatively low, reflecting the effectiveness of the existing donor selection and screening procedures. For example, studies have reported the prevalence of HBV, HCV, HIV, and syphilis among blood donors in Saudi Arabia to be in the range of 0.7%-1.2%, 0.4%-0.7%, 0.02%-0.03%, and 0.1%-0.25%, respectively [7]. Therefore, studying the prevalence of TTDs among blood donors in Makkah, Saudi Arabia, is essential for ensuring the safety of blood transfusions in the region and provides insights that can guide local healthcare policies and practices. In addition, understanding the prevalence of TTDs can directly impact healthcare costs, with the aim to reduce morbidity and long-term health complications. Early detection and exclusion of infected donations can avert these costs and contribute to the efficiency and sustainability of healthcare services.

Several methods are currently being deployed for the detection of these diseases. Nucleic acid testing (NAT) allows for the direct detection of viral genomes in blood donations and is commonly used for HIV, HCV, and HBV screening [8]. Enzyme-linked immunosorbent assay (ELISA) is frequently employed to screen blood donations for antibodies or antigens related to HIV, HBV, HCV, HTLV-1, and syphilis [9]. Rapid diagnostic tests (RDTs) and serological testing are quick and cost-effective and are utilized for HIV, HCV, syphilis, and malaria screening and for detecting antibodies or antigens in the blood, respectively [9]. In this study, we will rely on these techniques with the aim of studying the prevalence of infectious diseases such as HBV, HCV, HIV, malaria, HTLV-1, and syphilis among blood donors in the Central Blood Bank in Makkah, Saudi Arabia.

## Materials and methods

Research design and sample collection

**Figure 1:**
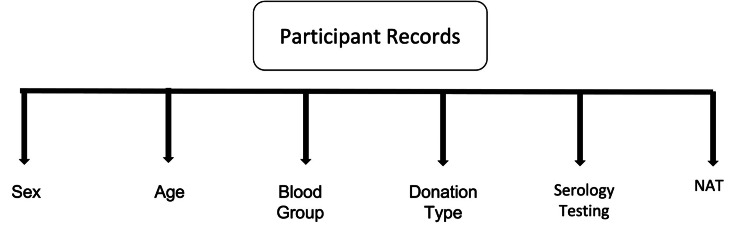
Participants' records NAT: nucleic acid testing

This is a retrospective study that includes 7,875 participants from the Central Blood Bank in Makkah, Saudi Arabia, from January 1, 2022, to December 30, 2022. These records, as mandated by Saudi blood donation protocols, provide a comprehensive dataset encompassing each donor's demographic characteristics, medical history, and information regarding any past infectious diseases. The process of data collection is strategically designed to prioritize donor health and safety, by ensuring the systematic exclusion of individuals with known histories of infection or chronic medical conditions. The inclusion criteria for this study are adherence to Saudi protocols for blood donation, and the donor should be eligible for participation, be in optimal health, be free from infectious diseases, age between 18 and 65 years, weigh a minimum of 50 kg, and meet certain other physiological benchmarks, such as specified ranges of hemoglobin, pulse rate, temperature, and blood pressure. However, to ensure the integrity and completeness of the dataset, only donors with complete records were included in the analysis (Figure 1). This measure will reduce potential sources of error and bias associated with missing or incomplete data.

Donor laboratory testing

The Central Blood Banks adhere to rigorous national screening guidelines to ensure the safety and efficacy of donated blood units. The testing process includes serological examinations using an automated chemiluminescent microparticle immunoassay analyzer, the ARCHITECT i2000 system (CMIA, Abbott Diagnostic, USA). This system, in conjunction with validated assays, allows for the detection of several key health markers. In addition to this, the presence of *Treponema pallidum*, the bacterium responsible for syphilis, is determined via an initial screening process using the Immulite-2000 XPi system (Siemens Healthcare Diagnostic Products Ltd., Surrey, UK). Reactive samples are subjected to further confirmatory tests to validate the infection status.

Ethical approval 

Ethical approval for this study was taken from the Ethics Committee of the Ministry of Health, Saudi Arabia, and the Institutional Review Board in the Central Blood Banks in Makkah (approval number: H-02-K-076-0123-868).

Statistical analysis

The data was entered into a Microsoft Excel spreadsheet (Microsoft Corporation, Redmond, WA) before being analyzed using the Statistical Package for the Social Sciences (SPSS) software version 22.0 (IBM SPSS Statistics, Armonk, NY). This analysis consists of calculating frequencies and percentages and establishing potential correlations using the Pearson correlation coefficient in the data. Group comparisons were conducted using the chi-square test, with a p-value of less than 0.05 considered statistically significant.

## Results

Sociodemographic characteristics of the blood donors

A total of 7,875 blood units were received and tested at Central Blood Bank in Makkah, Saudi Arabia, from January 2022 to December 2022. Descriptive statistics, variables, and outcomes for these samples are shown in Table 1. Among the 7,875 total blood donors, the majority were male, representing 99.5% (7,838) of the total donors. This gender disparity may stem from various societal, cultural, and demographic factors. As shown in Table 1, the ages of the donors varied, but noticeably, there were no donors under the age of 18 or over the age of 60, each of these age groups accounting for 0% of the total donor population. The age group with the largest representation was those aged between 29 and 39 years, who made up 46.9% (3,695) of the total donors. This was followed by those aged 18-28 years, who accounted for 32.9% (2,591) of the donors. Donors aged 40-50 years comprised 18.2% (1,437) of the total, while those aged 51-60 years made up a smaller portion, representing 1.9% (152) of the total donor pool. In terms of blood type, O+ve was the most common, found in 40.3% (3,177) of donors, followed by A+ve in 25.3% (1,989) of donors. The other blood types, B+ve, B-ve, A-ve, AB+ve, AB-ve, and O-ve, were found in 21.6% (1,701), 1.5% (115), 0.5% (39), 5.5% (436), 1.9% (153), and 3.4% (265) of donors, respectively. As for the reasons for donation, the majority of donors, 74.1% (5,832), donated as a replacement for a patient. Donors who gave blood as part of obtaining a driving license represented 21.3% (1,681) of the total, while voluntary donors made up 4.6% (362) of the donors (Table 1).

**Table 1:** Sociodemographic characteristics of the blood donors

Characteristic parameter	Subcategory	Number of units	Percentage	Total number of blood donors (cumulative)
Sex	Male	7,838	99.5%	7,875
	Female	37	0.5%	
Age (years)	<18	0	0%	7,875
	18-28	2,591	32.9%	
	29-39	3,695	46.9%	
	40-50	1,437	18.2%	
	51-60	152	1.9%	
	>60	0	0%	
Blood type	A-ve	39	0.5%	7,875
	A+ve	1,989	25.3%	
	B-ve	115	1.5%	
	B+ve	1,701	21.6%	
	AB-ve	153	1.9%	
	AB+ve	436	5.5%	
	O-ve	265	3.4%	
	O+ve	3,177	40.3%	
Donation type	Voluntary	362	4.6%	7,875
	Replacement to patient	5,832	74.1%	
	Driving license	1,681	21.3%	

Prevalence of infectious serological markers in the blood units

The prevalence of infectious serological markers in the blood units was explored (Table 2). Hepatitis B virus surface antigen (HBsAg), a marker for hepatitis B virus infection, was found to be non-reactive in the vast majority of the cohort (99.6%), signifying a lack of current infection. However, a small portion (0.4%) was reactive, indicating potential current infection. Anti-HB antibody was predominantly non-reactive (93.2%), with a minor section (6.8%) showing reactivity. Further, results showed that hepatitis B core antibody (HBcAb) was non-reactive in 92.5% of the samples and was reactive in 7.5%. This antibody generally presents in both acute and chronic hepatitis B virus infections. Hepatitis C virus antibody (HCV Ab) reactivity was noted in a small fraction (0.7%) of the cohort, whereas the majority (99.3%) was non-reactive. Turning toward HIV I/II antigen/antibody (Ag/Ab), the study found that 99.9% of the samples were non-reactive, suggesting no current infection with HIV, while a negligible portion (0.1%) was reactive. Similar results were obtained for the human T-lymphotropic virus I/II antibody (HTLV I/II Ab), with only 0.1% of samples reactive. Finally, the nucleic acid testing (NAT) used for viral detection showed a non-reactive outcome in 99.6% of the samples. However, 0.1% were reactive for HCV (R-HCV), 0.4% were reactive for HBV (R-HBV), and a negligible portion (0%) was reactive for HIV (R-HIV) (Table 2).

**Table 2:** Prevalence of infectious serological markers and NAT in the blood units HBsAg: hepatitis B virus surface antigen, anti-HB Ab: anti-hepatitis B virus antibody, HBcAb: hepatitis B virus core antibody, HCV Ab: hepatitis C virus antibody, HIV I/II Ag/Ab: hepatitis C virus I/II antigen/antibody, HTLV I/II Ab: human T-lymphotropic virus I/II antibody, NAT: nucleic acid testing, R-HCV: reactive for HCV, R-HBV: reactive for HBV, R-HIV: reactive for HIV

Parameter	Status	Number of units	Prevalence (%)	Total number of blood units
HBsAg	Non-reactive	7,847	99.6%	7,875
	Reactive	28	0.4%	
Anti-HB Ab	Non-reactive	7,345	93.2%	7,875
	Reactive	530	6.8%	
HBcAb	Non-reactive	7,285	92.5%	7,875
	Reactive	590	7.5%	
HCV Ab	Non-reactive	7,820	99.3%	7,875
	Reactive	55	0.7%	
HIV I/II Ag/Ab	Non-reactive	7,866	99.9%	7,875
	Reactive	9	0.1%	
HTLV I/II Ab	Non-reactive	7,867	99.9%	7,875
	Reactive	8	0.1%	
NAT	Non-reactive	7,840	99.6%	7,875
	R-HCV	6	0.1%	
	R-HBV	28	0.4%	
	R-HIV	1	0%	

Correlation of seroprevalence of anti-hepatitis B virus surface antibodies (HBsAb), HCV antibodies (Ab), and HIV antibodies (Ab) in relation to nucleic acid testing (NAT)

**Figure 2:**
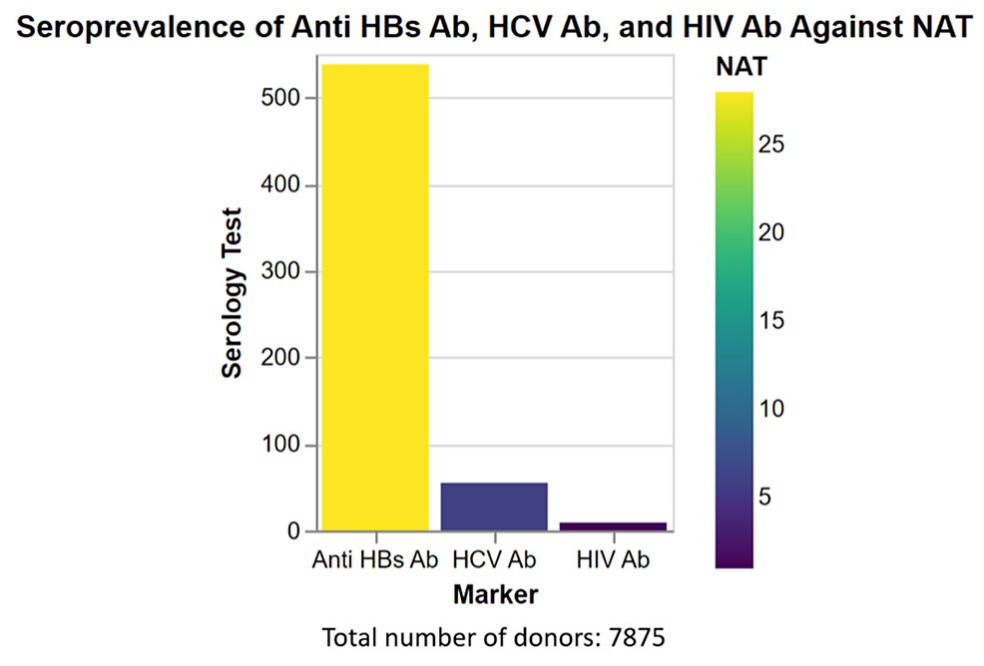
Seroprevalence of anti-HBsAb, HCV Ab, and HIV Ab against NAT The x-axis represents the marker of anti-HBsAb, HCV Ab, and HIV Ab, and the y-axis represents the NAT serology test. The color of the bars indicates the NAT percentage of each status. HBsAb: hepatitis B virus surface antibody, HCV Ab: hepatitis C virus antibody, HIV I Ab: hepatitis C virus I antibody, NAT: nucleic acid testing

The seroprevalence of anti-hepatitis B virus surface antibodies (HBsAb), HCV antibodies (Ab), and HIV antibodies (Ab) in relation to nucleic acid testing (NAT) were assessed as shown in Figure 2. Among the samples tested, 538 individuals tested positive for anti-HBsAb, while 28 samples tested positive for NAT. For HCV Ab, 55 individuals tested positive, with six samples showing positive results for NAT. In the case of HIV Ab, nine individuals tested positive, and one sample tested positive for NAT. These findings provide valuable information about the seroprevalence of these markers and their correlation with NAT, which is crucial for blood screening and the management of infectious diseases.

The Pearson correlation analysis reveals a strong positive linear relationship between serology reactive and NAT-positive results, as indicated in Table 3, by a correlation coefficient of 0.995. This nearly perfect positive correlation suggests that as the serology reactive count increases, the NAT-positive results similarly increase. However, the significance level (p-value) of this relationship is 0.061, which is slightly above the commonly accepted threshold of 0.05. Thus, this result would not typically be deemed statistically significant, meaning we do not have sufficient evidence to reject the null hypothesis of no association between the two variables.

**Table 3:** Correlation analysis between anti-HBsAb, HCV Ab, and HIV Ab against NAT HBsAb: hepatitis B virus surface antibody, HCV Ab: hepatitis C virus antibody, HIV Ab: hepatitis C virus antibody, NAT: nucleic acid testing

	Pearson correlation	p-value
Serology	1	0.061
NAT	0.995	0.061

A comparative analysis of hepatitis B markers (HBsAg and anti-HBsAb) among a sample of 7,875 blood donors was done as indicated in Table 4. Out of the total 7,847 units, there was around 99.64% non-reactive to HBsAg, suggesting no active hepatitis B infection, while 28 (0.36%) units were reactive, indicating an active hepatitis B infection. In terms of immunity to hepatitis B, as measured by anti-HBsAb, 7,337 (93.17%) units were non-reactive, suggesting an absence of immunity, whereas 538 (6.83%) units were reactive, indicating either past exposure to the virus or vaccination.

**Table 4:** Comparative prevalence of HBsAg and anti-HBsAb among blood donors HBsAg: hepatitis B virus surface antigen, anti-HBsAb: anti-hepatitis B virus surface antibody

Marker	Status	Number of units	Percentage	Total number of blood units tested
HBsAg	Non-reactive	7,847	99.64%	7,875
HBsAg	Reactive	28	0.36%	7,875
Anti-HBsAb	Non-reactive	7,337	93.17%	7,875
Anti-HBsAb	Reactive	538	6.83%	7,875

Table 5 provides a correlation matrix that examines the relationships between each pair of variables: Total_no_units, HBsAg, and HBsAb. Starting with the relationship between Total_no_units and HBsAg, a Pearson correlation coefficient of 0.030 is observed. This suggests a very weak positive relationship between these variables, implying that as the total number of units tested increases, the non-reactivity or reactivity to HBsAg slightly increases. This relationship is statistically significant at the 0.01 level given its p-value of 0.008.

**Table 5:** Correlation analysis between anti-HBsAg and anti-HBsAb HBsAg: hepatitis B virus surface antigen, HBsAb: hepatitis B virus surface antibody

	Pearson correlation	p-value
HBsAg	0.030	0.008
Anti-HBsAb	-0.437	0.001
HBsAg and HBsAb	-0.016	0.151

The correlation between Total_no_units and HBsAb yields a Pearson correlation coefficient of -0.437, signifying a moderate negative correlation. This suggests that as the total number of units tested increases, the non-reactivity or reactivity to HBsAb tends to decrease. This negative correlation is also statistically significant at the 0.01 level, as indicated by a p-value of less than 0.001.

Lastly, the relationship between HBsAg and HBsAb results in a Pearson correlation coefficient of -0.016, suggesting a very weak negative correlation. However, with a p-value of 0.151, this correlation is not statistically significant at the 0.01 level, indicating no substantial linear relationship between the reactivity statuses of HBsAg and HBsAb within this dataset.

## Discussion

This study aimed to highlight several key sociodemographic characteristics of blood donors in Makkah, Saudi Arabia, from January 2022 to December 2022. In particular, this study offers insights into the prevalence of various infectious diseases using serological testing and NAT. We also aimed to conduct a correlation analysis of HBsAg and anti-HBsAb, markers critical to the detection and management of hepatitis B, one of the most significant bloodborne diseases globally. Data will give important insights into the safety of blood donation in Makkah and how current policies are effective.

Our data showed males representing 99.5% of the total donors. Studies from different countries support this finding and suggest some possible reasons for the gender disparity, such as social and cultural factors and physiological differences as females often experience periods of ineligibility due to low hemoglobin, menstruation, or pregnancy [10,11]. We showed that most blood donors are in the 29-39 years age group (46.9%), followed by the 18-28 years (32.9%) and the 40-50 years age groups (18.2%). These findings are similar to a study conducted in Nigeria and Uganda [12]. Age is a crucial determinant of blood donation, and we can see that most blood donors in this study were in the age of 29-39 years age group because younger people are usually healthier and more eligible.

The most common blood type of blood donors in our study was O+ve, consistent with many studies in which O+ was shown to be the most common blood type worldwide and in Saudi Arabia [13,14]. Furthermore, our data showed that the most common donation type of blood donors was those with replacement purposes, which aligned with a study from Ethiopia that showed a similar trend where replacement donors constituted most of the donor population [15].

Data showed that the main reason for donation was replacement to other patients. Giving blood as a volunteer in our cohort was uncommon. The importance of voluntary non-remunerated blood donors has been emphasized by the World Health Organization, as they are the safest group of donors with the lowest prevalence of bloodborne infections. Our data provides valuable insights into the sociodemographic profile of blood donors, and it highlights the need for more tailored recruitment strategies, especially for encouraging female and voluntary donors.

The presence of hepatitis B surface antigen (HBsAg), indicative of an active hepatitis B virus (HBV) infection, was found to be reactive in only 0.4%. This low prevalence might be attributed to the effective implementation of vaccination programs and the observance of infection control practices in Saudi Arabia. However, it remains a concern because HBV is a leading cause of liver disease worldwide. This result is consistent with the global decrease in HBV prevalence [16]. Anti-HBsAb were found to be reactive in 6.8%, implying a past or resolved HBV infection or a response to HBV vaccination. Hepatitis B core antibody (HBcAb), an indicator of past or present HBV infection, showed a prevalence of 7.5%, which is higher than that of HBsAg. This discrepancy may be because HBcAb remains detectable for a longer period, even after the disease is cleared [17]. Hepatitis C virus (HCV) antibodies were reactive in 0.7%, implying a past or present HCV infection. The prevalence of HCV has been reported to vary globally, with higher rates in regions with poor public health infrastructures and inadequate blood screening practices. This finding is lower than the global estimate of HCV prevalence (2.5%) [18]. For HIV I/II Ag/Ab and HTLV I/II Ab, both were found to be reactive in 0.1% of the total blood units, indicating a relatively low prevalence in the blood donor population. However, these diseases are of great concern given their significant morbidity and mortality. The low prevalence is consistent with global estimates and might be due to the stringent pre-donation screening and low prevalence in the general population [19]. Syphilis, detected by Venereal Disease Research Laboratory (VDRL) test, was reactive in 0.5%. This finding reflects the continuous efforts to control sexually transmitted infections but remains a concern due to the severity of the disease if left untreated.

Nucleic acid testing (NAT) for HIV, HBV, and HCV provides the most sensitive detection of these viruses. The reactive rates for R-HCV (0.1%), R-HBV (0.4%), and R-HIV (0%) in the NAT test suggest the effectiveness of this method in reducing the window period for the detection of these infections, thus enhancing blood safety [20]. The seroprevalence of anti-HBsAb, HCV Ab, and HIV Ab was identified and compared by another approach such as NAT. This comparison is essential as it highlights the sensitivity and specificity of each screening method, contributing to better strategies for blood screening and infectious disease management. For anti-HBsAb, there were 538 reactive results via serology testing, whereas only 28 were identified as positive through NAT. It is important to note that anti-HBsAb are antibodies produced by the immune system in response to the hepatitis B surface antigen. Their presence may indicate either a past infection that the immune system has effectively fought off or the effectiveness of hepatitis B vaccination. Hence, these antibodies' presence does not necessarily mean active infection, which could explain the discrepancy in the results between the two methods [21]. Regarding HCV Ab, there were 55 reactive results via serology testing, with only six positive results identified through NAT. Serology tests for HCV Ab identify antibodies produced in response to an HCV infection, signaling past or current infection. However, it is not uncommon for these antibodies to remain present in the blood, even when the virus is no longer active. NAT, on the other hand, identifies the actual viral RNA in the blood, offering more precise evidence of an active infection [22]. In terms of HIV Ab, there were nine reactive results via serology testing and one positive result through NAT. Similar to the aforementioned infections, the HIV serology test identifies antibodies against the virus. However, it may not be accurate during the early stage of infection (window period), or the antibodies may still be present even after the viral load becomes undetectable due to treatment or spontaneous clearance. NAT can more accurately identify active infections by detecting viral genetic material, hence the discrepancy.

The Pearson correlation analysis results in a strong correlation between the serology tests and NAT, with a correlation coefficient of 0.995. This high correlation suggests that the reactive results from the serology tests generally align with the NAT results, implying an increase in reactive serology results aligns with an increase in NAT positive results. The p-value, however, stands at 0.061, slightly higher than the accepted threshold of 0.05 for statistical significance, suggesting that the correlation, although strong, is not statistically significant.

In this study, the prevalence of hepatitis B markers (HBsAg and anti-HBsAb) among the tested blood donors was investigated. Our result indicates that only 0.36% were reactive for HBsAg and 6.83% were reactive for anti-HBsAb, indicating either past infection or vaccination in these individuals. These data suggested a low prevalence of active hepatitis B infections among these donors, and a large proportion (93.17%) of the tested samples were non-reactive for anti-HBsAb, suggesting a lack of immunity to hepatitis B among these donors. This could be concerning as it indicates susceptibility to infection and can have implications for future hepatitis B transmission and public health strategies, underlining the importance of enhancing hepatitis B vaccination coverage [23].

The correlation analysis expands on the relationship between HBsAg and anti-HBsAb. The Pearson correlation coefficient of 0.030 between the total number of units tested and HBsAg, although statistically significant (p-value of 0.008), suggests a very weak positive relationship.

The negative correlation between the total number of units tested and HBsAb (-0.437) is stronger and statistically significant (p-value of less than 0.001). This suggests that as more units were tested, the proportion of individuals showing immunity to hepatitis B (as evidenced by the presence of anti-HBsAb) decreased.

The correlation analysis between HBsAg and HBsAb showed a trend with a negative correlation, suggesting that those with an active hepatitis B infection (positive HBsAg) are less likely to have immunity (positive anti-HBsAb). However, this correlation was not statistically significant (p-value of 0.151), indicating no strong linear relationship between these two variables in this dataset. Thus, these results indicate a low prevalence of active hepatitis B infections but a similarly low prevalence of hepatitis B immunity among the studied population, suggesting the need for further interventions to increase hepatitis B vaccination coverage.

## Conclusions

In conclusion, this study provides crucial insights into the donor population's demographic characteristics and the prevalence of several serological markers, particularly those related to hepatitis B. These findings could guide the design of public health interventions aimed at increasing the safety of the blood supply and improving vaccination coverage for hepatitis B. In addition, our results highlight the importance of using complementary testing methods for blood screening to ensure both past and present infections are identified, contributing to improved blood safety and patient care.

## References

[1] Ackfeld T, Schmutz T, Guechi Y, Le Terrier C (2022). Blood transfusion reactions-a comprehensive review of the literature including a Swiss perspective. J Clin Med.

[2] Xiao H, Song W, Ai H (2023). Correlation between mortality and blood transfusion in patients with major surgery initially admitted to intensive care unit: a retrospective analysis. BMC Anesthesiol.

[3] Lan N, Stocchi L, Li Y, Shen B (2018). Perioperative blood transfusion is associated with post-operative infectious complications in patients with Crohn's disease. Gastroenterol Rep (Oxf).

[4] Panch SR, Savani BN, Stroncek DF (2019). Transfusion support in patients with hematologic disease: new and novel transfusion modalities. Semin Hematol.

[5] Bloch EM (2022). Transfusion-transmitted infections. Ann Blood.

[6] Kebede E, Getnet G, Enyew G, Gebretsadik D (2020). Transfusion transmissible infections among voluntary blood donors at Dessie blood bank, northeast Ethiopia: cross-sectional study. Infect Drug Resist.

[7] Abdel Gader AG, Osman AM, Al Gahtani FH, Farghali MN, Ramadan AH, Al-Momen AK (2011). Attitude to blood donation in Saudi Arabia. Asian J Transfus Sci.

[8] Aggarwal A, Mehta S, Gupta D, Sheikh S, Pallagatti S, Singh R, Singla I (2012). Dental students' motivations and perceptions of dental professional career in India. J Dent Educ.

[9] Al-Matary AM, Al Gashaa FA (2022). Comparison of different rapid screening tests and ELISA for HBV, HCV, and HIV among healthy blood donors and recipients at Jibla University Hospital Yemen. J Med Life.

[10] Okoroiwu HU, Asemota EA (2019). Blood donors deferral prevalence and causes in a tertiary health care hospital, southern Nigeria. BMC Health Serv Res.

[11] Birhaneselassie M (2016). Prevalence of transfusion-transmissible infections in donors to an Ethiopian blood bank between 2009 and 2013 and donation factors that would improve the safety of the blood supply in underdeveloped countries. Lab Med.

[12] Aneke JC, Okocha CE (2017). Blood transfusion safety; current status and challenges in Nigeria. Asian J Transfus Sci.

[13] Torun YA, Kaynar LG, Karakükcü C (2012). ABO and Rh blood group distribution in Kayseri Province, Turkey. Turk J Haematol.

[14] Bawazir WM (2022). Genotyping of Dombrock blood group system in blood donors from Saudi Arabia: a single-center study. Saudi Med J.

[15] Ataro Z, Urgessa F, Wasihun T (2018). Prevalence and trends of major transfusion transmissible infections among blood donors in Dire Dawa blood bank, eastern Ethiopia: retrospective study. Ethiop J Health Sci.

[16] (2022). Global, regional, and national burden of hepatitis B, 1990-2019: a systematic analysis for the Global Burden of Disease Study 2019. Lancet Gastroenterol Hepatol.

[17] Lok AS, McMahon BJ (2009). Chronic hepatitis B: update 2009. Hepatology.

[18] Brunner N, Bruggmann P (2021). Trends of the global hepatitis C disease burden: strategies to achieve elimination. J Prev Med Public Health.

[19] Gessain A, Ramassamy JL, Afonso PV, Cassar O (2023). Geographic distribution, clinical epidemiology and genetic diversity of the human oncogenic retrovirus HTLV-1 in Africa, the world's largest endemic area. Front Immunol.

[20] Wu D, Feng F, Wang X (2022). The impact of nucleic acid testing to detect human immunodeficiency virus, hepatitis C virus, and hepatitis B virus yields from a single blood center in China with 10-years review. BMC Infect Dis.

[21] (2017). EASL 2017 clinical practice guidelines on the management of hepatitis B virus infection. J Hepatol.

[22] Hans R, Marwaha N (2014). Nucleic acid testing-benefits and constraints. Asian J Transfus Sci.

[23] Ott JJ, Stevens GA, Groeger J, Wiersma ST (2012). Global epidemiology of hepatitis B virus infection: new estimates of age-specific HBsAg seroprevalence and endemicity. Vaccine.

